# Apple detection and instance segmentation in natural environments using an improved Mask Scoring R-CNN Model

**DOI:** 10.3389/fpls.2022.1016470

**Published:** 2022-12-02

**Authors:** Dandan Wang, Dongjian He

**Affiliations:** ^1^ College of Communication and Information Engineering, Xi’an University of Science and Technology, Xi’an, China; ^2^ Xi’an Key Laboratory of Network Convergence Communication, Xi’an, China; ^3^ College of Mechanical and Electronic Engineering, Northwest A&F University, Xianyang, China

**Keywords:** fruit, detection, segmentation, deep learning, Mask Scoring R-CNN, attention mechanism

## Abstract

The accurate detection and segmentation of apples during growth stage is essential for yield estimation, timely harvesting, and retrieving growth information. However, factors such as the uncertain illumination, overlaps and occlusions of apples, homochromatic background and the gradual change in the ground color of apples from green to red, bring great challenges to the detection and segmentation of apples. To solve these problems, this study proposed an improved Mask Scoring region-based convolutional neural network (Mask Scoring R-CNN), known as MS-ADS, for accurate apple detection and instance segmentation in a natural environment. First, the ResNeSt, a variant of ResNet, combined with a feature pyramid network was used as backbone network to improve the feature extraction ability. Second, high-level architectures including R-CNN head and mask head were modified to improve the utilization of high-level features. Convolutional layers were added to the original R-CNN head to improve the accuracy of bounding box detection (*bbox_mAP*), and the Dual Attention Network was added to the original mask head to improve the accuracy of instance segmentation (*mask_mAP*). The experimental results showed that the proposed MS-ADS model effectively detected and segmented apples under various conditions, such as apples occluded by branches, leaves and other apples, apples with different ground colors and shadows, and apples divided into parts by branches and petioles. The *recall*, *precision*, false detection rate, and *F*1 score were 97.4%, 96.5%, 3.5%, and 96.9%, respectively. A *bbox_mAP* and *mask_mAP* of 0.932 and 0.920, respectively, were achieved on the test set, and the average run-time was 0.27 s per image. The experimental results indicated that the MS-ADS method detected and segmented apples in the orchard robustly and accurately with real-time performance. This study lays a foundation for follow-up work, such as yield estimation, harvesting, and automatic and long-term acquisition of apple growth information.

## 1 Introduction

The production and management of apple orchards mainly rely on experienced growers, which has the disadvantages of being time-consuming, labour-intensive, high cost and low precision (Barbole et al., 2021). With the rapid development of precision and intelligent agriculture, machine vision has become an important way to obtain apple growth information. Apple detection and segmentation through machine vision is the foundation of an innovative orchard management method. It is of great significance for monitoring the growth and nutritional status of fruit, performing early yield estimation and timely harvesting, and it can effectively reduce the dependence on manual labour (Tian et al., 2019; Jia et al., 2020). However, the complex growth environment in orchards, fluctuating illumination, uneven distribution of fruits, overlaps and occlusions of apples, change of apple color during the growth process, varying colors and shadows on the surface of apples, and other environmental variables in the natural orchard have a significant impact on the accurate detection and segmentation of apples (Tang et al., 2020; Wang and He, 2022).

Many methods have been proposed to solve the problems mentioned above. For instance, Gongal et al. (2015) used histogram equalization first to intensify color differences between apples and background and then used Otsu threshold and edge detection methods to detect foreground pixels. Finally, Circular Hough Transformation and Blob detection were used to detect apples in images. The accuracy of this method was 82% with dual-side imaging. In another study, based on the color, texture, and three-dimension (3D) shape properties, Rakun et al. (2011) developed an apple image segmentation method, where color features and threshold segmentation were used to segment potential apple region from the background. Further, texture analysis and 3D reconstruction were utilized to refine the color-segmented area, and finally apple image segmentation were achieved. It is also believed that using artificial lighting during night time, a bright spot would appear on the surface of apple. Linker and Kelman (2015) used this property to design a method for detecting green apples, they found that this method was insensitive to the color of apples. These traditional image processing methods use manually designed features for target detection and segmentation. However, apple growth environment is complex, and the illumination conditions constantly change over time. Texture, shape and color features of fruit change due to light intensity, occlusions and overlaps. It is very difficult to extract the universal features of apples in natural environment, resulting in poor universality of traditional methods (Zhou et al., 2012; Nguyen et al., 2016; Fu et al., 2020).

With the development of machine learning, deep learning has been widely applied in the agricultural field (Tian H. et al., 2020; Naranjo-Torres et al., 2020; Saleem et al., 2021). Compared with traditional image processing methods, the deep learning-based methods avoid complex operations, such as image pre-processing and target feature extraction. These methods take images as input and extract appropriate features automatically (Guo et al., 2016). Deep learning achieves outstanding results with good robustness. Recently, it has been applied to fruit detection and segmentation (Jia et al., 2020; Maheswari et al., 2021; Jia et al., 2021; Jia et al., 2022a). For example, Kang and Chen (2019); Kang and Chen (2020) designed a detection and segmentation network (DaSNet) to achieve the accurate segmentation of apples. Li et al. (2021) proposed an ensemble U-Net segmentation model for immature green apple segmentation. To compensate for the poor performance of the deep convolutional neural network in keeping the edge of the target, the edge features of the apples were fused with the high-level features of U-Net (Ronneberger et al., 2015) to achieve accurate segmentation of the apples. The experimental results showed that this method ensured the segmentation accuracy of apples and improved the generalisation ability of the model. A suppression mask region-based convolutional neural network (R-CNN) was developed by Chu et al. (2021) to detect apples. In this study, a suppression branch was added to the standard Mask R-CNN (He et al., 2020), which effectively suppressed the generation of non-apple features and improved the accuracy of detection. To realize the accurate segmentation of green fruit, Jia et al. (2022b) proposed an efficient You Only Look One-level Feature (YOLOF)-snake segmentation model. In the research, the contour based instance segmentation method Deep-snake algorithm module is embedded after the YOLOF regression branch. The method achieved the fast and accurate segmentation of green fruit. Liu J. et al. (2022) proposed a DLNet model to detect and segment obscured green fruits. They introduced an approach consisting of a detection network and a segmentation network. In the detection network, the Gaussian non-local attention mechanism was added to the feature pyramid network (FPN) to build a refined pyramid network that could continuously refine semantic features generated by the residual network (ResNet) (He et al., 2016) and FPN. The segmentation network was composed of a dual-layer Graph Attention Network (GAT). The experimental results showed that this method has high accuracy in detecting and segmenting green fruits with good robustness. An obscured green apple detection and segmentation method based on a fully convolutional one-stage (FCOS) object detection model was proposed by Liu M. Y. et al. (2022). They used a residual feature pyramid to improve the detection accuracy of green fruits of various sizes and fused a two-layer convolutional block attention network into FCOS to recover the edges of incomplete green fruits. The accuracy of detection and segmentation were 77.2% and 79.7%, respectively. Compared with traditional methods, the accuracy and generalization ability of the above deep learning-based methods are significantly improved. However, most of the researches focus on immature green fruit or mature red fruit. The detection and segmentation of fruit whose ground color gradual change from green to red throughout the whole growth period in natural orchard remains a challenge. Currently, study on apple detection and segmentation based on deep learning is still under development, and there are few studies on the detection of apple in the whole growth periods. Additionally, the existing methods mainly focus on detecting fruit with little occlusion and simple lighting conditions (Jia et al., 2022c), which is difficult to meet the development needs of intelligent management of orchard.

Image segmentation includes semantic and instance segmentation. Semantic segmentation generates the same mask for the same class, rendering it ineffective in separating overlapping objects of the same class. Instance segmentation integrates object detection and segmentation and generates a different mask for each object. For apples grown in natural orchards, fruit overlap is common; hence instance segmentation is more applicable for apple detection and segmentation. Mask Scoring R-CNN (Huang et al., 2019) is one of the state-of-the-art instance segmentation methods, which is widely used in the detection and instance segmentation of various targets. For example, Tian Y. et al. (2020) applied Mask Scoring R-CNN to apple flower detection. They fused U-Net into Mask Scoring R-CNN, and proposed a MASU-R-CNN model. Tu et al. (2021) used Mask Scoring R-CNN to segment pig images, achieving the effective segmentation of adhesive pigs.

With the development of deep learning, the attention mechanism has gradually become an important component. Fusing the attention mechanism into network can effectively increase the expression ability of the network model and allows it to focus on important features of the target while suppressing unnecessary features (Zhu et al., 2019). Recently, attention mechanisms have also been used for fruit detection. Jiang et al. (2022) fused the non-local attention module (Wang et al., 2018) and convolutional block attention model, inspired by the Squeeze-and-excitation network (Hu et al., 2018), into a You Only Look Once (YOLO) V4 to achieve high-efficiency detection of young apples. The experimental results showed that the added attention module effectively improved the detection accuracy. Liu J. et al. (2022) added the Gaussian non-local attention mechanism to the FPN to refine the semantic features continuously generated by the ResNet and FPN.

The overall goal of this study is to provide a reliable and efficient method to detect and instance segment apples throughout the whole growth periods in complex environment. Inspired by the above successful researches, a method based on an improved Mask Scoring R-CNN (MS-ADS) that fused attention mechanism was proposed. Specific objectives are as follows:

1. To improve the feature extraction ability of the backbone, ResNeSt, a variant of ResNet fused with attention mechanism, combined with FPN was used to replace the original backbone network of the Mask Scoring R-CNN.
2. To further improve the utilization of high-level features and enhance the accuracy of bounding box detection and instance segmentation, the R-CNN head and mask head of the Mask Scoring R-CNN were improved by adding convolution layers and Dual Attention Network (DANet), respectively.
3. Train and test the MS-ADS model to achieve the accurate detection and instance segmentation of apples in the natural environment.

The MS-ADS method focus on reliable and efficient detection and segmentation of apples throughout the whole growth stages. The method was achieved by improving the backbone and high-level architectures including R-CNN head and mask head of the original model. The improvement of backbone allows the network to improve its feature extraction ability by being more attentive to the apple features and effectively ignoring background features. High-level feature maps, containing rich context and semantic information, are useful in determining the invariant and abstract features that could be used for a variety of vision tasks including target detection and classification. By modifying high-level architectures, it was conducive to improving the utilization of high-level features to obtain more accurate detection results and more refined edge segmentation results. Accurate apple detection and segmentation throughout the growth period are crucial for realizing yield estimation, timely harvesting and automatic monitoring of the fruit growth. The proposed method can be used to count the growth cycles of apple, and simultaneously perform appropriate variable rate irrigation and fertilization according to the monitored growth state or density of the fruits at different growth stages, which then improves the resource utilization efficiency. Additionally, this method can also provide a reference for storage facilities according to production estimation.

## 2 Materials and methods

### 2.1 Image dataset acquisition

In this study, apple images were captured in an experimental apple orchard belonging to the College of Horticulture, Northwest A&F University, Yangling, Shaanxi, China. The images used in this research were collected from 9:00 to 11:00 a.m. and 3:00 to 6:30 p.m. from May to September in 2019 during cloudy and sunny weather conditions. Images under natural daylight with backlight and direct sunlight conditions were acquired using an iPhone 7 Plus. The images were captured with a resolution of 4032 × 3024 pixels and were saved in JPEG format.

To improve computational efficiency and to adapt to the images collected by cameras with a low resolution, the images were rescaled to 369 × 277 pixels. To make the edges of the apple clearer and facilitate image annotation and subsequent feature extraction, the images were sharpened using the Laplace operator (Gonzalez & Woods, 2020). The rescaled and sharpened images were manually annotated by polygons using the VGG image annotator (VIA) (Dutta and Zisserman, 2019) for network training and testing. After annotating, 219 images acquired under various conditions were selected as the test set, and the remaining images were used as the training set. **Table 1** shows the information of the apple dataset.

**Table 1:** Information of apple dataset.

Conditions	Color of apple	Number of training set/Number of annotated apples	Number of test set/Number of annotated apples	Total
Direction sunlight	Red	173/428	35/68	208/496
	Green	191/513	41/74	232/587
	Uneven	187/479	36/65	223/544
Backlight	Red	151/441	35/67	186/508
	Green	160/536	35/73	195/609
	Uneven	159/458	37/74	196/532
Total		1021/2855	219/421	1240/3276

### 2.2 Apple detection and instance segmentation based on the improved Mask Scoring R-CNN (MS-ADS)

**Figure 1:**
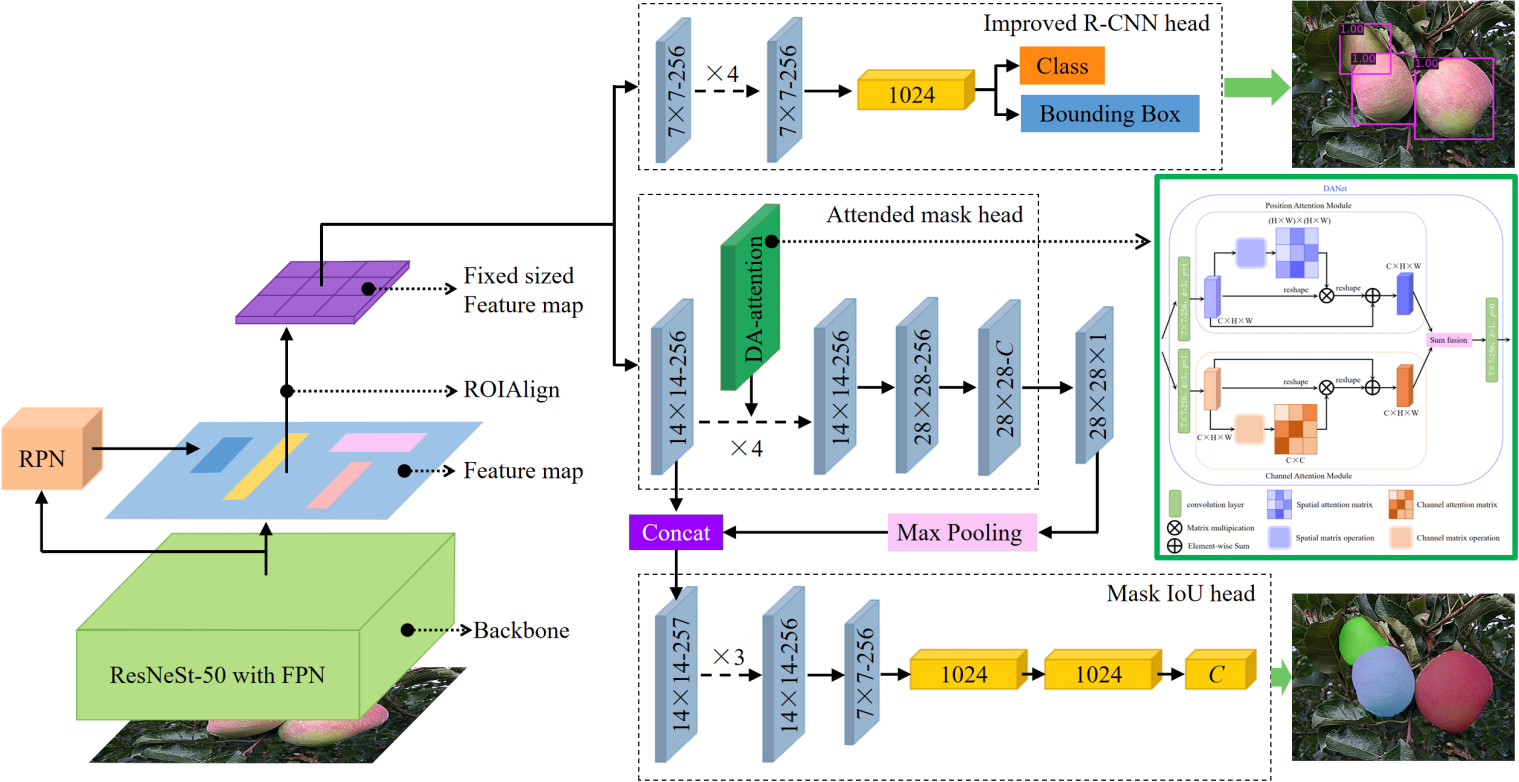
Structure of MS-ADS network. 1. Input images into backbone for feature extraction. 2. Input obtained feature maps into RPN and RoIAlign. 3. Fed acquired feature maps into R-CNN head for classification and bounding box regression, and into attended mask head and Mask IoU head for apple instance segmentation.

Mask Scoring R-CNN is one of the state-of-the-art detection and instance segmentation methods. It is improved from the Mask R-CNN (He et al., 2020) by adding a maskIoU branch to achieve accurate object detection and instance segmentation. In this study, an MS-ADS network model based on an improved Mask Scoring R-CNN was proposed to accurately detect and segment apples in orchards. **Figure 1** shows the structure of the MS-ADS network, which includes three parts: (1) Backbone network: ResNeSt (Zhang et al., 2022), which is a variant of ResNet, combined with FPN, was used as the backbone network for extracting features of the images. (2) The output of the backbone network was fed into the region proposal network (RPN) to generate the region proposals. Then, RoIAlign extracted features from each proposal to properly align the features with the input. (3) Classification and bounding box regression of apples were performed, and the masks of apples were generated.

#### 2.2.1 Backbone network of MS-ADS

A backbone network is used to extract features from images for subsequent object detection and segmentation. In this study, ReNeSt-50, a variant of ResNet-50 fused with attention mechanism, combined with FPN, was used as the backbone network.

ResNeSt network (Zhang et al., 2022), which improves based on ResNet, combines the advantages of Squeeze-and-Excitation networks (Hu et al., 2018), Selective Kernel networks (Li et al., 2019), and ResNeXt (Xie et al., 2017). As in ResNeXt blocks, in ResNeSt blocks, a Cardinality hyperparameter is given to divide the feature map into *K* groups. Meanwhile, a radix hyperparameter is defined to divide each group into *R* splits. Then, the input *X* is divided into *G* groups, *G* = *KR*, and *X* = {*X*_1_, *X*_2_,…, *X_G_*}. A series of transformations *F* = {*F*_1_, *F*_2_,…, *F_G_*} are performed on each individual group, then the intermediate representation of each group is *U_i_* = *F_i_* (*X_i_*), *i* ∈{1, 2,…, *G*}. A weighted fusion of the cardinal group representation *V^k^* ∈*ℝ^H^* ^×^ *^W^* ^×^ *^C^* ^/^ *^K^* (*H*, *W* and *C* are the sizes of output feature map) is aggregated using channel-wise soft attention, where each feature map channel was produced using a weighted combination of over splits. The features of the *c*-th channel are calculated by the formula (1).


(1) $$V_{c}^{k}=\sum i{=}_{1}^{R}a_{i}^{k}(c)U_{R(K-1)+i}$$

where $a_{i}^{k}(c)$ denotes an assignment weight. The cardinal group representations are then concatenated along the channel dimension: *V*= Concat{*V* ^1^, *V* ^2^,…, *V^K^*}. In a standard residual block, if the input and output feature map share the same shape, the final output *Y* of the ResNeSt block is produced using a shortcut connection: *Y* = *V* + *X*. For blocks with a stride, the shape of the input and output feature map are not the same; hence, an appropriate transformation *T* is applied to the shortcut connection to align the output shapes: *Y* = *V* + *T*(*X*).

**Figure 2:**
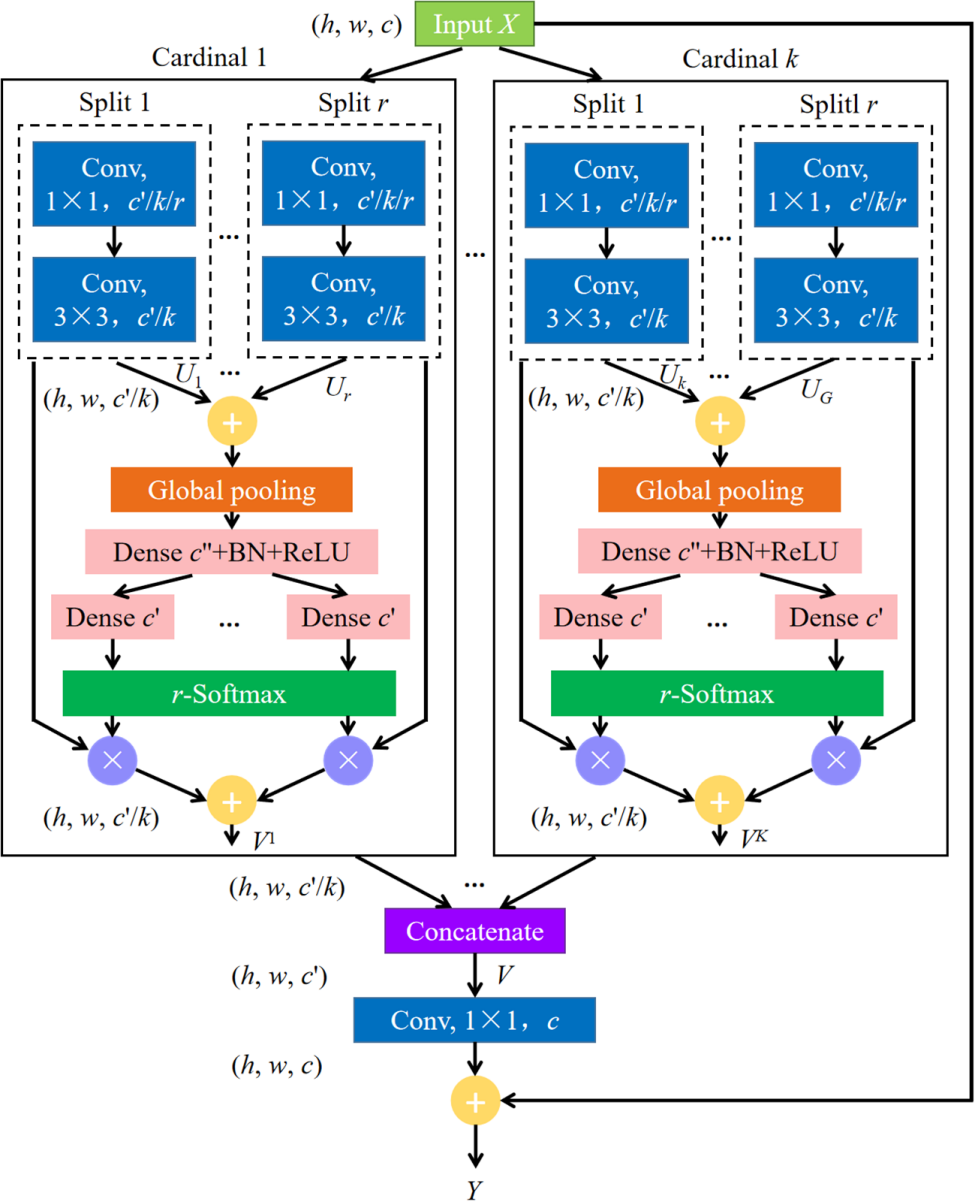
ResNeSt block. *r* is the number of Splits. *r* = 1, 2,…, *R*. *k* denotes the number of Cardinal, *k* = 1, 2,…, *K*. *h*, *w* and *c* represent the height, width and channel of the input feature map, respectively. Conv represents convolutional layer, and Global pooling means global average pooling. BN and ReLU are batch normalization and activation function, respectively.

The ResNeSt block is shown in **Figure 2**. An equivalent transformation of network model shown in **Figure 2** was used in this experiment for it can be modularized and accelerated by group convolution and standard CNN layers (Zhang et al., 2022). In this study, we used ResNeSt-50 to extract features. The parameter *R* was set to 2, and *K* was set to 1. The output of ResNeSt-50 was used as the input for FPN and together they functioned as the backbone network of our MS-ADS model. FPN extracts multi-scale features from a pyramid hierarchy of convolutional neural networks and combines the features of each stage of the ResNeSt-50 network to give network semantic and spatial information, thus improving its accuracy.

#### 2.2.2 Generation of Region of interest and RoIAlign

The feature maps generated by the backbone network were fed into RPN to search RoIs where apples are located. When generating RoIs, according to the actual situation of a single fruit on the image, three area scales, including 32 × 32, 64 × 64 and 128 × 128, and three aspect ratios as 1:1, 1:2 and 2:1 were randomly combined to generate nine anchors. The anchors were used to predict the location of apples to enhance the accuracy of the RoI outputs. After generating RoIs, the RoIs and the corresponding feature maps were input into RoIAlign to adjust the size of the anchor box to a fixed size. RoIAlign properly aligned the extracted features with the input to improve the pixel-level segmentation accuracy.

#### 2.2.3 Apple detection and instance segmentation based on MS-ADS

The feature maps obtained from RoIAlign were used as input for the high-level heads of MS-ADS model. The heads included an improved R-CNN head, an attended mask head and a Mask IoU head. High-level feature maps, containing rich context and semantic information, are useful in determining the invariant and abstract features that could be used for a variety of vision tasks including target detection and classification. By modifying high-level architectures (R-CNN head and mask head), it was conducive to improving the utilization of high-level features to detect apples of various scales. Improving high-level architectures could be necessary and beneficial for obtaining more accurate detection results and more refined edge segmentation results.

##### 2.2.3.1 Improved R-CNN head

The improved R-CNN head of the MS-ADS model, which was used for classification and bounding box regression, was composed of convolutional layers and a fully connected layer. The structure of the improved R-CNN head is shown in **Figure 1**. Four convolutional layers were added to the original R-CNN head to extract features sufficiently and improve the accuracy of the final classification and regression. The kernel size, padding and stride of the added convolutional layers were 3 × 3, 1 and 1, respectively, and the output channel was 256.

##### 2.2.3.2 Attended mask head

**Figure 3:**
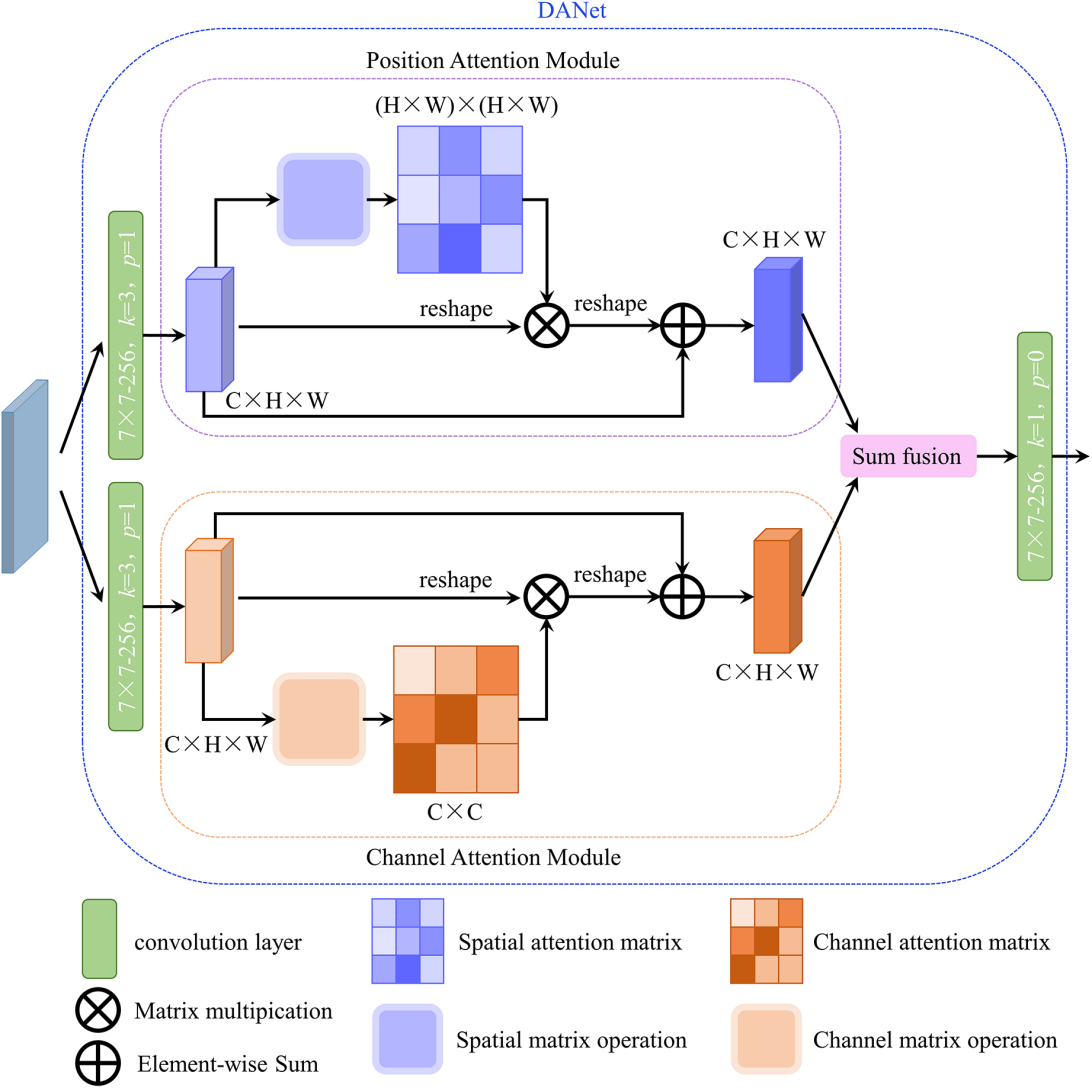
Structure of DANet. (*H*, *W* and *C* are the height, width and channel of the input feature map, respectively).

To further improve the accuracy of instance segmentation, in this research, the DANet (Fu et al., 2019) was inserted into the original mask head. The structure of DANet is illustrated in **Figure 3**. DANet draws global context over local features, including a position attention module and a channel attention module. The position attention module selectively integrates the feature at each position through a weighted sum of the features at all positions (similar features would be related to each other, regardless of their distances). The channel attention module selectively emphasizes interdependent channel maps by aggregating relevant features among all channel maps. DANet sums the outputs of the two attention modules to further enhance the feature representation and to achieve more accurate segmentation results.

In this study, DANet was inserted followed by the second convolutional layers of the original mask head (as shown in **Figure 1**) to get a precise segmentation mask. The improved mask head was named as attended mask head.

##### 2.2.3.3 MaskIoU head

MaskIoU head consists of convolutional layers and fully connected layers. It regresses the IoU between the predicted mask and its ground truth mask. The output features of the RoIAlign and the predicted mask were concatenated, and the concatenation result was used as the input for the MaskIoU head. The output of the MaskIoU head is the number of classes. In this study, the number of classes is 1, that is, the apple class.

#### 2.2.4 Loss function

The loss function represents the difference between the prediction and the ground truth, which is very important in network training. The loss function of the MS-ADS network model was composed of two parts: RPN loss and the training loss of the three heads, as shown in formula (2).


(2) $$L=L_{RPN}+L_{heads}$$

where *L* is the loss of the MS-ADS network model, *L_RPN_* is the loss of RPN, and it can be calculated by the formula (3).


(3) $$L_{RPN}=\frac{1}{N_{RPN\_cls}}\sum i_{L}^{RPN\_cls}(p_{i,}p_{i}^{*})+\lambda \frac{1}{N_{RPN\_box}}\sum p_{i}^{*}L_{RPN\_box}(t_{i},t_{i}^{*})$$

where, *L_RPN_*___ *_cls_* and *L_RPN_*___ *_bbox_* are the classification loss and the bounding box regression loss of RPN, respectively. λ is a balance parameter. *N_RPN_*___ *_cls_* and *N_RPN_*___ *_bbox_* are the mini-batch size and the number of anchor locations, respectively. *P_i_* is the classification probability of anchor *i*, and $p_{i}^{*}$ is the ground truth label probability of anchor *i*. *t_i_* represents the difference between the predicted bounding box and the ground truth labelled box. $t_{i}^{*}$ denotes the difference between the ground truth labelled box and the positive anchor.

*L_heads_* represents the loss of the three heads, and it is a sum of the loss of the three heads. *L_heads_* can be calculated by the formula (4).


(4) $$L_{heads}=L_{cls}+L_{box}+L_{mask}+L_{maskIoU}$$

where, *L_cls_* and *L_bbox_* are the classification loss and the bounding box regression loss of the improved R-CNN head, respectively, *L_mask_* is the mask loss of attended mask head, and *L_maskIoU_* is the MaskIoU loss of MaskIoU head. The loss function of the three heads in this study is the same as those of the original Mask Scoring R-CNN.

#### 2.2.5 Network training and evaluation of MS-ADS network model

The processor used in this study was an Intel Core i7-7700HQ, with a 16 GB RAM and an 8 GB NVIDIA GTX 1070 GPU. We trained the network on Ubuntu 16.04, and Python 3.6 was used in the training and testing of the MS-ADS network model.

The original Mask Scoring R-CNN model pre-trained on the COCO dataset (Lin et al., 2014) was used to initialize the MS-ADS to accelerate the training process. The manually annotated apple images were then utilized for training and testing the MS-ADS network. The iteration number was set to 24 epochs. The initial learning rate was set to 0.02 and later decreased by ten times at the 16th and 22nd epochs, respectively. The momentum and weight decay were set to 0.9 and 1 × 10^−4^, respectively. The total training time lasted for 3 h and 6 min.

To test the performance of the proposed MS-ADS method on the detection and instance segmentation of apples, *precision*, *recall, F*1 score, mean average precision of the detection bounding box (*bbox_mAP*), mean average precision of the segmentation mask (*mask_mAP*) and average run time were used to evaluate the method.

## 3 Results

### 3.1 Apple detection and instance segmentation using the MS-ADS method

**Figure 4:**
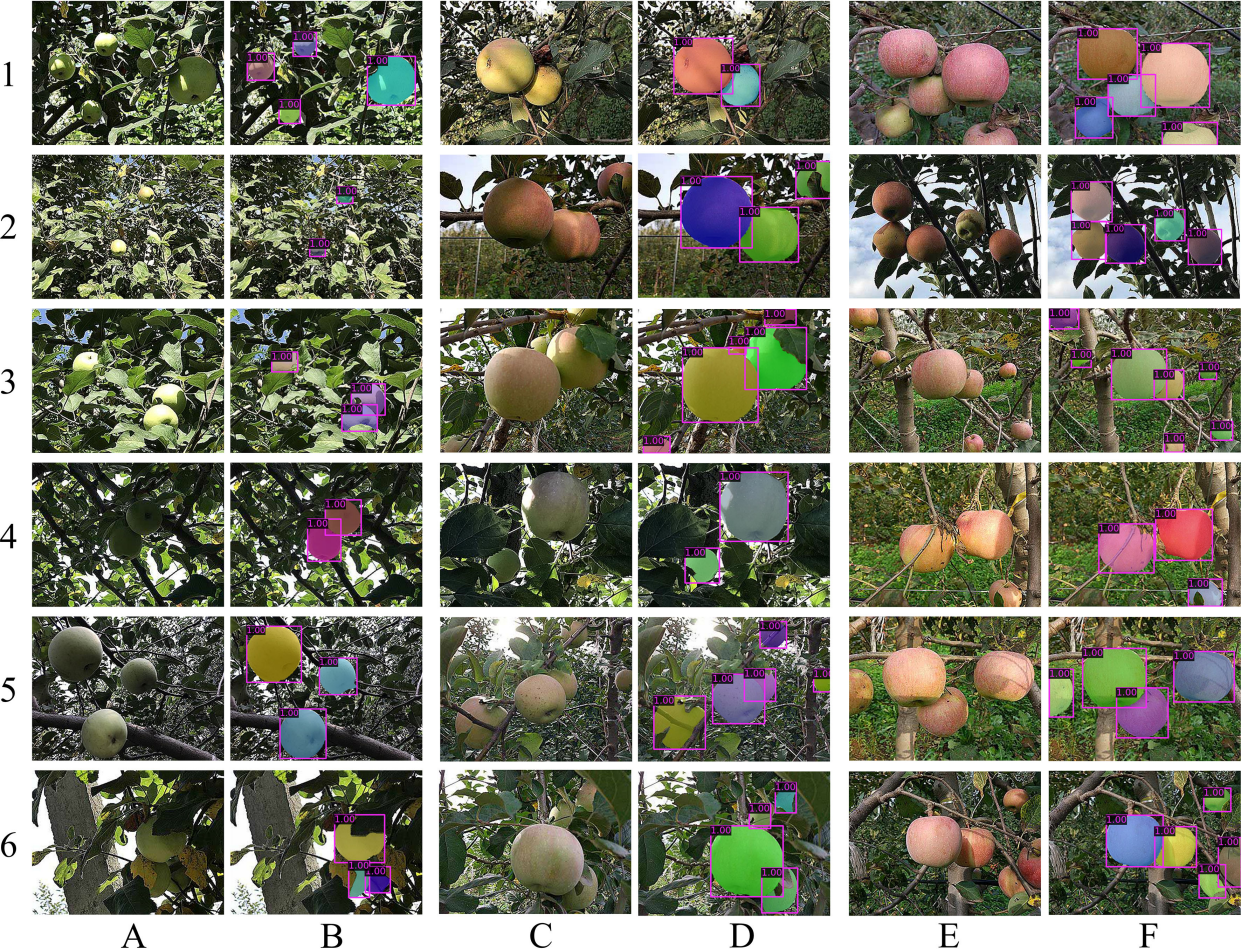
Examples of detection and instance segmentation of apples. **(A, C, E)** Original images. Specifically, (A1) Green apples affected by shadows. (A2) Small green apples with strong illumination on the surface. (A3) Apples affected by overlap, occlusion, shadows, and strong illumination. (A4) Green apple image captured under backlight condition. (A5) Green apples with uneven illuminations on the surface. (A6) Green apples with high similarities to the background. (C1) Overlapped apples with uneven colors. (C2) Apples affected by occlusion, shadows, and uneven colors. (C3) Apples affected by overlap, occlusion, shadows, and uneven colors. (C4) Apples with uneven colors and shadows on the surface captured under backlight conditions. (C5) Apples affected by overlap, occlusion, uneven colors, and backlight. (C6) Apples affected by overlap, occlusion, and uneven colors. (E1) Red overlap apples and apples with uneven colors. (E2) Red apples with uneven illuminations and apples with uneven colors. (E3) Overlapped and small red apples. (E4) Red apples affected by occlusion and shadows. (E5) Red apples affected by overlap and shadows. (E6) Red apples affected by overlap, occlusion, and shadows. (B1-6, D1-6, F1-6) Detection and instance segmentation results of images in **(A, C, E)**.

To verify the effectiveness of the proposed MS-ADS method, 219 apple images captured during the growth stage were used to test the method. The *precision* and *recall* of the MS-ADS method were 96.5% and 97.4%, respectively, and the false detection rate was 3.5%. The *bbox_mAP* and *mask_mAP* were 0.932 and 0.920, respectively, on the test set, and the average run time was 0.27 s (**Table 2**). Examples of the detection and instance segmentation results are illustrated in **Figure 4**. To further analyze the detection results of apples under various conditions, the recall of apples affected by different factors, such as independent apples, occluded apples, apples divided into parts by branches and petioles, clustered apples, red apples, green apples, apples with uneven colors, shadows and uneven illumination on the surface, were calculated and analyzed. The results are shown in **Table 3**.

**Table 2:** Detection and instance segmentation results of apples on test set.

Evaluations	*precision/*%	*recall/*%	*F1*/%	*False detection*/%	*bbox_ mAP*	*mask_mAP*	run time/s
MS-ADS	96.5	97.4	96.9	3.5	0.932	0.920	0.27

**Table 3:** Detection results of apples under different conditions.

Conditions	IA	OA	DA	CA	RA	GA	UC	SA	UI
*recall/*%	98.9	96.3	95.8	96.6	98.3	96.8	97.5	97.2	98.1

IA, Independent apple; OA, Occluded apples; DA, Apples divided into parts by branches and petioles; CA, Clustered apples; RA, Red apples; GA, Green apples; UC, Apples with uneven colors on the surface; SA, Apples with shadows on the surface; UI, Apples with uneven illuminations on the surface.

As can be seen in **Figure 4** and **Table 3**, the MS-ADS method was accurate and effective in detection and instance segmentation of green apples (**Figure 4A**), apples with uneven colors on the surface (**Figure 4C**) and red apples (**Figure 4E**), and the detection recall of these apples were 98.3%, 96.8%, and 97.5%, respectively. The MS-ADS method achieved accurate detection for apples occluded by branches and leaves (**Figures 4A1, A3, A6, C3, C5, C6, E4, E6**), and the detection recall was 96.3%. For apples occluded by branches and leaves, detection of apples divided into multiple parts by branches or petioles (**Figures 4A6, C5, C6, E4**) are often considered a special case. It is relatively difficult to detect this kind of apple. However, the detection recall of apples under this condition using the MS-ADS method was 95.8%, indicating that the proposed method is applicable for the detection and segmentation of apples divided into parts by branches or petioles. The MS-ADS method was also effective in detecting clustered apples (**Figures 4A3, A4, A6, C1, C2, C3, C5, C6, E1, E2, E3, E5, E6**), and the detection recall was 96.6%. Apples with shadows (**Figures 4A1, A3, C2, C3, C4, E4, E5, E6**) and uneven illumination (**Figures 4A5, E2**) on the surface were also accurately detected by the MS-ADS method. The detection recall of apples with shadows and uneven illumination on the surface were 97.2% and 98.1%, respectively. Additionally, the detection results for apples with extremely strong illumination (**Figures 4A2, A3**), extremely dark illumination on the surface (**Figure 4A4**) and extremely small apples (**Figures 4A2, E3**) by the MS-ADS method were all satisfactory. The MS-ADS method was also effective in detecting apples that were similar to the backgrounds (**Figure 4A6**), a task that is difficult even for human eyes.

From the detection and instance segmentation results shown in **Figure 4**, **Tables 2** and **3**, it is clear that the proposed MS-ADS method overcame the effect of colors, illuminations, overlap, occlusion, complex background and shadows, and accurately and effectively detected and segmented apples under various conditions with good robustness.

### 3.2 Comparison with other methods

To further analyze the performance of the proposed MS-ADS method, parameters including *precision*, *recall*, *F*1 score, *bbox_mAP*, *mask_mAP*, and average run time, were used to evaluate the MS-ADS method. The performance of the method was compared with that of other six methods: YOLACT (Bolya et al., 2019), PolarMask (Xie et al., 2020), Mask R-CNN (He et al., 2020) with ResNet-50-FPN as backbone, Mask R-CNN with ConVeXt-T (Liu Z. et al., 2022) as backbone, Mask R-CNN integrated with GRoIE (Rossi et al., 2021), and Mask Scoring R-CNN (Huang et al., 2019). The configurations used in the seven methods are shown in **Table 4**. In the comparison experiments, 5-fold cross-validation was used to evaluate the seven methods. We divided the dataset into 5 parts: 219, 256, 255, 255, and 255 to make the ratio of training set to test set was about 8:2 in each experiment. **Table 5** gives the detection and instance segmentation results of the seven methods, and the results was the average of the five independent experiments.

**Table 4:** Configurations of seven methods.

Methods	Backbone	Initial learning rate	Momentum	Weight decay	Iteration number
Mask R-CNN	ResNet-50-FPN	0.02	0.9	1 × 10^−4^	24 epochs
Mask R-CNN	ConVeXt-T	0.00007	0.9	5 × 10^−2^	24 epochs
Mask Scoring R-CNN	ResNet-50-FPN	0.02	0.9	1 × 10^−4^	24 epochs
YOLACT	ResNet-50-FPN	0.001	0.9	5 × 10^−4^	55 epochs
PolarMask	ResNet-50-FPN	0.01	0.9	1 × 10^−4^	12 epochs
Mask R-CNN + GRoIE	ResNet-50-FPN	0.02	0.9	1 × 10^−4^	12 epochs
MS-ADS	ResNeSt-50-FPN	0.02	0.9	1 × 10^−4^	24 epochs

As can be seen from **Table 5**, the proposed MS-ADS method was more accurate in apple detection in terms of precision, F1 score, and bbox_mAP compared with the other six methods. Although the recall and mask_mAP for apples were lower than those of ConVeXt-T-based Mask RCNN, MS-ADS had a faster detection and segmentation speed and smaller computation than ConVeXt-T-based Mask RCNN. Though the run time was longer than that of methods including YOLACT, PolarMask, Mask R-CNN (ResNet-50-FPN) and Mask Scoring R-CNN, MS-ADS method was more accurate in detecting and segmenting apples throughout the whole apple growth period. Through the above comparison and analysis, the MS-ADS method outperformed other six methods, which enabled real-time and accurate detection and segmentation of apples under complex background.

**Table 5:** Detection and instance segmentation results of seven methods.

Methods	Evaluation parameters/Average					
	*precision/*%	*recall/*%	*F*1/%	*bbox_ mAP*	*mask_mAP*	run time/s
Mask R-CNN (ResNet-50)	92.8	94.5	93.6	0.919	0.908	0.17
Mask R-CNN (ConVeXt-T)	95.9	**97.0**	**96.4**	0.925	**0.920**	0.39
Mask Scoring R-CNN	94.4	95.8	95.1	0.921	0.910	0.25
YOLACT	91.5	92.9	92.2	0.891	0.905	**0.16**
PolarMask	92.0	93.5	92.7	0.908	0.903	0.21
Mask R-CNN +GRoIE	94.8	96.3	95.5	0.923	0.908	0.40
**MS-ADS**	**96.0**	96.9	**96.4**	**0.928**	0.918	0.29

The best values are marked bold.

## 4 Discussion

### 4.1 Analysis of detection and segmentation results of apples in the growth period

Accurate fruit detection and segmentation during the growth period are crucial for realizing yield estimation, timely harvesting and automatic monitoring of the fruit growth. Apples are grown in open and unstructured orchards; therefore, the detection and segmentation of apples are affected by several factors, such as the fluctuating illumination, overlapping and occlusion of apples and similarities between immature green apples and the background color, which makes accurate detection and segmentation of apples challenging. An MS-ADS method was proposed in this study to solve these problems. To further improve the detection and segmentation accuracy of the Mask Scoring R-CNN model, a ResNeSt, which is a variant of ResNet fused with attention mechanism, combined with FPN, was used to replace the backbone network of the original Mask Scoring R-CNN. This allowed the network to improve its feature extraction capability by being more attentive to the apple features and effectively ignoring background features. Convolutional layers were added to the original R-CNN head to improve the accuracy of bounding box regression. Simultaneously, a dual attention network was inserted into the original mask head to improve the segmentation accuracy. The apple detection and instance segmentation results of the MS-ADS method showed that this method accurately detected and segmented apples under various conditions in a real-time way.

**Figure 5:**
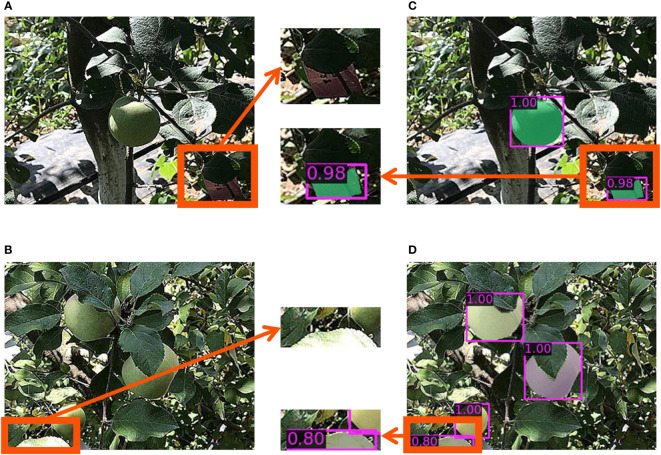
False detection and segmentation. **(A, B)** Original images. **(C, D)** Detection and instance segmentation results of original images **(A, B)**.

There were also false detection and segmentation when using the MS-ADS method, as shown in **Figure 5**. False detection was mainly caused by the high similarities between the background and apples. As shown in **Figure 5A**, a tag, which was made by testers, was falsely detected as an apple. In the image shown in **Figure 5B**, a green leaf was falsely detected as an apple. Future improvements will be made by expanding training samples with similar backgrounds to reduce false detection. The false detection rate of the MS-ADS method in this study was 3.5%. Although there was false detection, the MS-ADS method achieved optimal detection and segmentation on the test set.

### 4.2 Effect of the improved parts of the model on apple detection and segmentation

**Figure 6:**
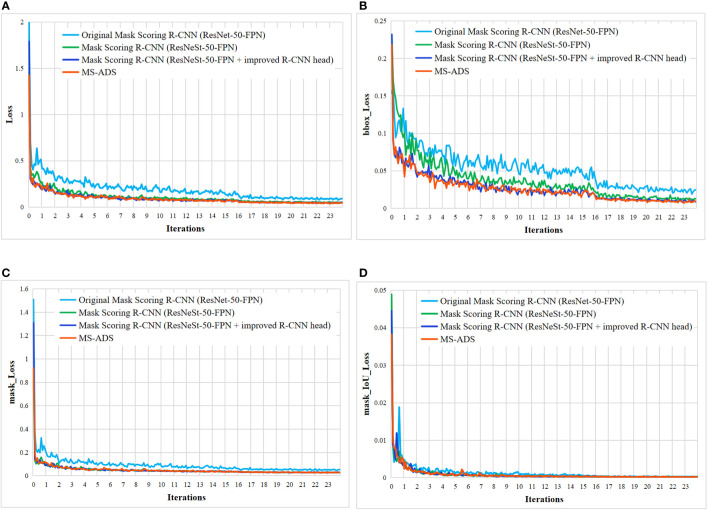
Loss curves of four methods. **(A)** Overall loss curves. **(B)** Bounding box loss curves. **(C)** Mask loss curves. **(D)** Mask_IoU loss curves.

**Figure 7:**
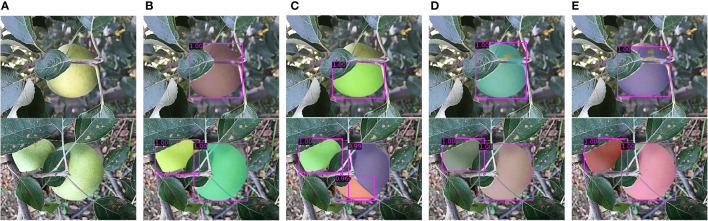
Comparison of detection and segmentation results of four methods. **(A)** Original images. **(B)** Results of the MS-ADS method. **(C)** Results of the original Mask Scoring R-CNN (ResNet-50-FPN). **(D)** Results of the Mask Scoring R-CNN with ResNeSt-50-FPN. **(E)** Results of the Mask Scoring R-CNN with ResNeSt-50-FPN and improved R-CNN head.

The proposed MS-ADS method was improved by modifying the Mask Scoring R-CNN (Huang et al., 2019). Firstly, the ResNeSt-50 combined with FPN was used as the backbone network to improve the feature extraction ability of the network. To further improve the accuracy of bounding box regression and segmentation, convolutional layers were added to the original R-CNN head to make feature extraction more sufficient, and DANet was inserted into the original mask head to make segmentation more accurate. To analyze the effect of each improvement on the performance of apple detection and segmentation, the training loss function (**Figure 6**), model parameters (**Table 6**) and the detection and segmentation results on 219 test images (**Table 6** and **Figure 7**) of the original Mask Scoring R-CNN (ResNet-50-FPN), Mask Scoring R-CNN with ResNeSt-50-FPN as the backbone network, Mask Scoring R-CNN with ResNeSt-50-FPN as the backbone network and improved R-CNN head, and the MS-ADS were compared.

**Table 6:** Detection and instance segmentation results of three methods.

Methods	Model size/MB	GFLOPs	Parameters	*precision/*%	*recall/*%	*F*1/%	*bbox_mAP*	*mask_mAP*	Train/h
Original Mask Scoring R-CNN (ResNet-50-FPN)	**481.4**	**85.4**	**60.0M**	94.9	96.4	95.6	0.924	0.915	**2.5**
Mask Scoring R-CNN (ResNeSt-50-FPN)	496.8	93.1	62.3M	96.5	97.1	96.8	0.924	0.919	2.7
Mask Scoring R-CNN (ResNeSt-50-FPN and improved R-CNN head)	507.3	207.8	65.4M	95.6	97.4	96.5	0.930	0.918	3.0
**MS-ADS**	510.4	207.8	63.6M	**96.5**	**97.4**	**96.9**	**0.932**	**0.920**	3.1

The best values are marked bold.

As can be seen from **Figure 6**, the training loss curve of the proposed MS-ADS model is lower than that of the other three models. We improved the backbone network, R-CNN head and mask head of the original Mask Scoring R-CNN. This, in turn, improved the quality of the generated bounding box and mask, and the overall loss was reduced in comparison to the other three models.

During the experiment, the original Mask Scoring R-CNN based on ResNet-50 was first used to conduct the experiment. In order to further improve the feature extraction ability of the backbone network, ResNeSt-50, a variant of ResNet-50, was used to replace the ResNet-50. From the detection and segmentation results of the two models, it can be found that although the model size, calculations and the number of parameters increased, the detection and segmentation results of the Mask Scoring R-CNN based on ResNeSt-50 had dramatically improved (concluded from the comparison of *precision*, *recall*, *F*1 score, *bbox_mAP* and *mask_mAP* of the two methods). To make the detection more accurate and improve the *bbox_mAP*, we added four convolutional layers in the R-CNN head to extract features sufficiently. From the experimental results, we observed that although the *bbox_mAP* had been improved, *precision* and *mask_mAP* were reduced. To further improve *precision* and *mask_mAP* and ensure a high *bbox_mAP*, DANet was inserted into the mask head. The experimental results showed that *bbox_mAP* and *mask_mAP* had improved, and *precision* rebounded after the addition of DANet. However, since we replaced the backbone of the original Mask Scoring RCNN, added convolutional layers in the RCNN head and inserted DANet in the mask head, the proposed model was more complicated than the original model and the computation had dramatically increased, which resulting in longer training time and detection time. The results, as shown in **Table 6**, indicate that although the model size, calculations, parameters and training time of the proposed MS-ADS method increased, the accuracy of the detection and segmentation had significantly improved, indicating that the MS-ADS model was suitable for the accurate detection and instance segmentation of apples in this study.

**Figure 7** shows the comparison results of the four methods. Although apples in images were detected and segmented by the four methods (i.e., the *precision* and *recall* were high), the quality of the detected bounding box (*bbox_mAP*) and the segmented mask (*mask_mAP*) were very different. By contrast, the MS-ADS method achieved accurate detection and segmentation of apples on the premise of ensuring the quality of bounding box detection and segmentation.

## Conclusions

The MS-ADS method was proposed in this study to accurately detect and instance segment apples in different growth stages. The method was developed from the original Mask Scoring R-CNN. First, ResNeSt-50, a variant of ResNet-50 fused with attention mechanism, combined with FPN, was used to replace the backbone network of the original Mask Scoring R-CNN to enhance the feature extraction ability of the network model. Second, convolutional layers were added to the original R-CNN head to make feature extraction more sufficient and further enhance the accuracy of the generated bounding box. Finally, the DANet was inserted into the original mask head to further improve the accuracy of instance segmentation. Compared with the original Mask Scoring R-CNN, the proposed MS-ADS model performed better at detecting and segmenting the apples under various conditions.

The MS-ADS method effectively and accurately detected and segmented apples under various conditions during the growth stage with good robustness and real-time performance. The *recall*, *precision*, *F*1 score, *bbox_mAP*, *mask_mAP* and the average run-time of our method were 97.4%, 96.5%, 96.9%, 0.932, 0.920 and 0.27 s per image, respectively, on test set. This research could provide a reference for developing an automatic and long-term monitoring system for retrieving apple growth information.

The detection and instance segmentation results of this method were an improvement on prior studies; however, the network model was relatively large, and many aspects still need improvement. In the future, we will continue to track the latest research results and further expand the training set to cover more kinds of apples and apples under various conditions. We will continue to study methods that can further streamline the network model and improve its efficiency and the accuracy of the detection and segmentation of apples.

## Data availability statement

The raw data supporting the conclusions of this article will be made available by the authors, without undue reservation.

## Author contributions

WD: Conceptualization, data curation, Methodology, Software, Formal analysis, Resources, Writing–original draft, Supervision, Funding acquisition. HD: Conceptualization, Writing–review and editing. All authors contributed to the article and approved the submitted version.

## Funding

This work was funded by the Natural Science Basic Research Program of Shaanxi (2022JQ-186); Talent introduction Program of Xi’an University of Science and Technology (2050121002).

## Conflict of interest

The authors declare that the research was conducted in the absence of any commercial or financial relationships that could be construed as a potential conflict of interest.

## Publisher’s note

All claims expressed in this article are solely those of the authors and do not necessarily represent those of their affiliated organizations, or those of the publisher, the editors and the reviewers. Any product that may be evaluated in this article, or claim that may be made by its manufacturer, is not guaranteed or endorsed by the publisher.
